# Hidden Links Between Skin Microbiome and Skin Imaging Phenome

**DOI:** 10.1093/gpbjnl/qzae040

**Published:** 2024-06-07

**Authors:** Mingyue Cheng, Hong Zhou, Haobo Zhang, Xinchao Zhang, Shuting Zhang, Hong Bai, Yugo Zha, Dan Luo, Dan Chen, Siyuan Chen, Kang Ning, Wei Liu

**Affiliations:** College of Life Science and Technology, Huazhong University of Science and Technology, Wuhan 430074, China; National Engineering Research Center for Nanomedicine, Huazhong University of Science and Technology, Wuhan 430074, China; Key Laboratory of Molecular Biophysics of the Ministry of Education, Hubei Key Laboratory of Bioinformatics and Molecular Imaging, Center of Artificial Intelligence Biology, Department of Bioinformatics and Systems Biology, Huazhong University of Science and Technology, Wuhan 430074, China; College of Life Science and Technology, Huazhong University of Science and Technology, Wuhan 430074, China; College of Life Science and Technology, Huazhong University of Science and Technology, Wuhan 430074, China; Key Laboratory of Molecular Biophysics of the Ministry of Education, Hubei Key Laboratory of Bioinformatics and Molecular Imaging, Center of Artificial Intelligence Biology, Department of Bioinformatics and Systems Biology, Huazhong University of Science and Technology, Wuhan 430074, China; College of Life Science and Technology, Huazhong University of Science and Technology, Wuhan 430074, China; College of Life Science and Technology, Huazhong University of Science and Technology, Wuhan 430074, China; College of Life Science and Technology, Huazhong University of Science and Technology, Wuhan 430074, China; Key Laboratory of Molecular Biophysics of the Ministry of Education, Hubei Key Laboratory of Bioinformatics and Molecular Imaging, Center of Artificial Intelligence Biology, Department of Bioinformatics and Systems Biology, Huazhong University of Science and Technology, Wuhan 430074, China; College of Life Science and Technology, Huazhong University of Science and Technology, Wuhan 430074, China; Key Laboratory of Molecular Biophysics of the Ministry of Education, Hubei Key Laboratory of Bioinformatics and Molecular Imaging, Center of Artificial Intelligence Biology, Department of Bioinformatics and Systems Biology, Huazhong University of Science and Technology, Wuhan 430074, China; National Engineering Research Center for Nanomedicine, Huazhong University of Science and Technology, Wuhan 430074, China; National Engineering Research Center for Nanomedicine, Huazhong University of Science and Technology, Wuhan 430074, China; Research Institute for Biomaterials, Tech Institute for Advanced Materials, College of Materials Science and Engineering, Suqian Advanced Materials Industry Technology Innovation Center, NJTech-BARTY Joint Research Center for Innovative Medical Technology, Nanjing Tech University, Nanjing 211816, China; College of Life Science and Technology, Huazhong University of Science and Technology, Wuhan 430074, China; Key Laboratory of Molecular Biophysics of the Ministry of Education, Hubei Key Laboratory of Bioinformatics and Molecular Imaging, Center of Artificial Intelligence Biology, Department of Bioinformatics and Systems Biology, Huazhong University of Science and Technology, Wuhan 430074, China; College of Life Science and Technology, Huazhong University of Science and Technology, Wuhan 430074, China; National Engineering Research Center for Nanomedicine, Huazhong University of Science and Technology, Wuhan 430074, China

**Keywords:** Skin phenome, Skin microbiome, Metagenome, Machine learning, Imaging

## Abstract

Despite the skin microbiome has been linked to skin health and diseases, its role in modulating human skin appearance remains understudied. Using a total of 1244 face imaging phenomes and 246 cheek metagenomes, we first established three skin age indices by machine learning, including skin phenotype age (SPA), skin microbiota age (SMA), and skin integration age (SIA) as surrogates of phenotypic aging, microbial aging, and their combination, respectively. Moreover, we found that besides aging and gender as intrinsic factors, skin microbiome might also play a role in shaping skin imaging phenotypes (SIPs). Skin taxonomic and functional α diversity was positively linked to melanin, pore, pigment, and ultraviolet spot levels, but negatively linked to sebum, lightening, and porphyrin levels. Furthermore, certain species were correlated with specific SIPs, such as sebum and lightening levels negatively correlated with *Corynebacterium matruchotii*, *Staphylococcus capitis*, and *Streptococcus sanguinis*. Notably, we demonstrated skin microbial potential in predicting SIPs, among which the lightening level presented the least error of 1.8%. Lastly, we provided a reservoir of potential mechanisms through which skin microbiome adjusted the SIPs, including the modulation of pore, wrinkle, and sebum levels by cobalamin and heme synthesis pathways, predominantly driven by *Cutibacterium acnes*. This pioneering study unveils the paradigm for the hidden links between skin microbiome and skin imaging phenome, providing novel insights into how skin microbiome shapes skin appearance and its healthy aging.

## Graphical Abstract

**Graphical Abstract:**
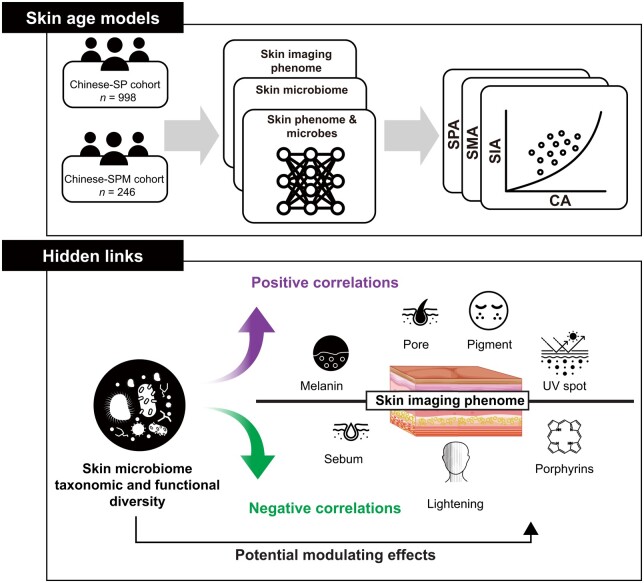

## Introduction

Skin health could be characterized by skin phenome and microbiome, as well as their interactions [1–3]. On the one hand, skin health could be directly reflected by the skin phenome, including physiological and molecular features such as moisture, pH, and lipid content [4,5], as well as imaging phenotypes such as lightening, wrinkle, texture, and pore [6–10]. On the other hand, another essential and underlying property of the skin, skin microbiome, has also been strongly correlated with the skin health, which exhibits the capability of fortifying the skin barrier and modulating the immune system [2,11,12], but phylogenetic and metabolic diversity with temporal stability [2,13,14]. Previous studies have reported the correlations of skin microbiome with skin diseases and physiological features [3,5,15]. However, the interactions between skin imaging features and skin microbiome remain understudied.

A few skin imaging phenotypes (SIPs) have been correlated with specific microbes. Spots, for instance, are reported to be produced by members of *Streptomyces glaucescens* and genus *Malassezia* [16–18]. Additionally, a randomized, double-blinded, placebo-controlled trial has shown that daily intake of *Lactobacillus plantarum* HY7714 could alleviate facial wrinkles [19]. A skin microbiota study based on 16S ribosomal RNA (rRNA) gene sequencing reported that the occurrence of Propionibacteriales, Corynebacteriales, and Bacillaceae orders was associated with improved skin texture, during the treatment of a skincare product [20]. In recent years, advanced sequencing technologies and computational methods in metagenomics have enabled the resolution of microbiome composition at deeper levels and the discovery of functional potential [21–24]. A metagenome study of large-scale cohorts with comprehensive skin features recorded may open new avenues for systematically exploring “how the microbiome influences human appearance”.

**Figure 1:**
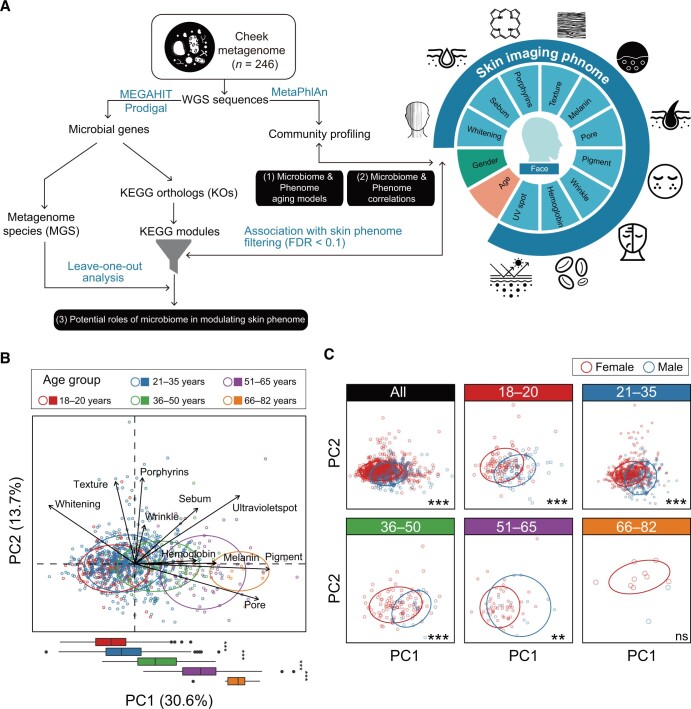
Intrinsic effects of age and gender on skin phenomes **A**. Workflow of the metagenome–phenome correlation study for the Chinese-SPM cohort. Skin metagenomes and SIPs were collected from a total of 246 females. The analysis contained two pipelines including using MetaPhlAn to obtain the microbial compositions and using MEGAHIT and Prodigal to construct the non-redundant gene catalog for MGS construction and functional annotations. The former pipeline aimed to answer the correlations between skin microbiomes and SIPs, and the latter aimed to explore how microbiomes influence SIPs. **B**. A PCA biplot based on skin imaging phenomes of samples (*n* = 998) in the Chinese-SP cohort, with the loadings of ten skin phenotypic vectors presented. Samples are grouped and colored according to age: 18–20 years (*n* = 154), 21–35 years (*n* = 662), 36–50 years (*n* = 109), 51–65 years (*n* = 62), and 66–82 years (*n* = 11). The ellipses represent the 80% confidence interval. The box plot shows the distribution differences of samples against the PC1 axis, and the statistical significance was tested using the Mann–Whitney–Wilcoxon test (*, *P* < 0.05; **, *P* < 0.001; ***, *P* < 0.001; ns, not significant). **C**. PCA plots based on skin imaging phenomes of samples in the Chinese-SP cohort, grouped and colored by gender. In each of the age groups, the differences in distribution against PC1 between male and female samples were tested. SIP, skin imaging phenotype; PCA, principal component analysis; Chinese-SPM, Chinese-skin-phenome-microbiome; Chinese-SP, Chinese-skin-phenome; MGS, metagenome species; WGS, whole-genome sequencing; KEGG, Kyoto Encyclopedia of Genes and Genomes; FDR, false discovery rate; KO, KEGG ortholog; UV, ultraviolet.

Here, we recruited two cohorts in a Chinese campus: (1) Chinese-skin-phenome (Chinese-SP) cohort encompassing 998 individuals’ skin imaging phenomes; and (2) Chinese-skin-phenome-microbiome (Chinese-SPM) cohort encompassing 246 cheek metagenomes and skin imaging phenomes. We established skin age indices as proxies of individual skin states in phenotypic and microbial aspects. Moreover, we investigated the correlations of microbial compositions and functions with skin imaging phenome. Furthermore, we identified the metagenome species (MGS) that most drove the modulatory effects of the skin microbiome on specific SIPs (**Figure 1**A), by establishing the MGS-SIP framework. Our work for the first time systematically showcased the intricate interactions between skin microbiome and skin imaging phenome.

## Results

To establish a skin phenome resource of healthy Chinese people, we recruited a total of 1000 individuals to test their face SIPs, and 998 of those aged 18–82 (29.08 ± 10.79) years constituted the Chinese-SP cohort after excluding two samples with abnormal phenotypic scores. For the microbiome study, we recruited another cohort of 246 females aged 20–60 (30.14 ± 9.21) years and examined their skin metagenomes in addition to their skin SIPs. Ten SIPs were collected by skin imaging technology [6–10], including lightening, sebum, porphyrins, texture, melanin, pore, pigment, wrinkle, hemoglobin, and ultraviolet (UV) spot (Figure S1). To fairly exclude the environmental effects on individual skin differences, all the individuals have been living on a campus in Wuhan, China. Moreover, the individuals had no skin diseases or skin disease history such as vitiligo, rosacea, psoriasis, or acne. All the individuals were required not to use any skincare products 24 h before the investigation.

### Temporal dynamics of skin imaging phenome

Using the Chinese-SP cohort, we identified the temporal dynamics of the skin phenome. Pigment, pore, UV spot, melanin, hemoglobin, and wrinkle levels rose with age, while lightening and texture levels declined [false discovery rate (FDR *<* 0.1, linear regression] (Figure S2). Sebum and porphyrins showed no significant correlation with age. To further depict the patterns of such temporal dynamics, we projected samples of the Chinese-SP cohort on a principal component analysis (PCA) plot (Figure 1B) and divided them into five subgroups based on their ages (year): 18–20, 21–35, 36–50, 51–65, and 66–82. Five age groups were separated against the PC1 axis (*P <* 0.001, Mann–Whitney–Wilcoxon test), with pigment, pore, and UV as the top vectors in an orientation of being older, while lightening as the top vector in an orientation of being younger. After the age of 18 years, all samples had a rise of the pigment, pore, and UV spot levels, but a decline of the lightening level, which persisted for at least 60 years (*P <* 0.05, Mann–Whitney–Wilcoxon test) (Figure S3A). Nevertheless, wrinkle formation and texture loss mainly occurred at the age of 36 years (*P <* 0.01, Mann–Whitney–Wilcoxon test). Furthermore, we discovered that gender was a strong confounder in the temporal dynamics of the SIPs, by comparing female and male samples. The overall skin phenotypic profiles differed between males and females across all the age groups in the 18–65 age range (*P <* 0.01, Mann–Whitney–Wilcoxon test) (Figure 1C). Moreover, females had higher wrinkle levels in the 18–20 age range (*P =* 6.29 × 10^−5^, Mann–Whitney–Wilcoxon test) (Figure S3B), and showed lower pigment, pore, and sebum levels but higher lightening levels in the 18–65 age range (*P <* 0.05, Mann–Whitney–Wilcoxon test). Together, these results reveal the temporal dynamics of skin imaging phenome and the intrinsic roles of age and gender in shaping human skin phenome.

### Establishment of the skin age indices as the proxies of skin imaging phenome and microbiome

**Figure 2:**
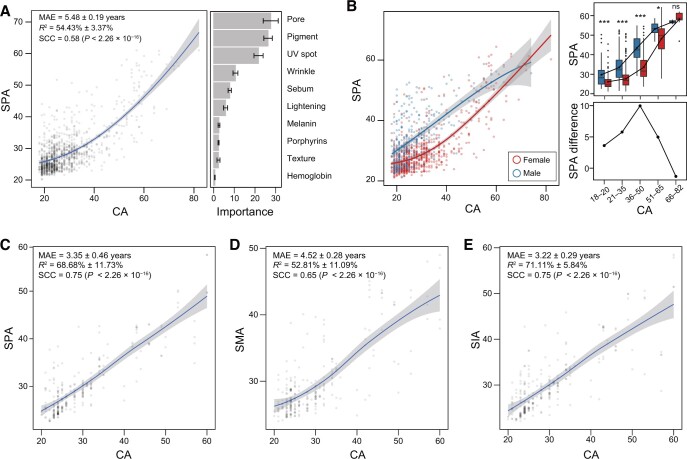
Skin age indices based on skin imaging phenome and skin microbiome **A**. RF regression to generate SPAs of the Chinese-SP cohort (*n* = 998) based on their SIPs. The bar plot with error bars shows feature importance ± SD of the ten SIPs in RF regression, using five-fold cross-validation. MAE refers to the mean absolute error between SPAs and CAs. **B**. RF regression to generate SPAs of females (*n* = 712) of the Chinese-SP cohort based on their SIPs, and then applied the trained model of females on male samples (*n* = 286). Box plot and line chart show the differences in SPAs across five groups of CAs. The differences in SPAs reflect the differences in the skin phenotypic states between males and females. For the box plot, statistical significance was tested using the Mann–Whitney–Wilcoxon test (*, *P <* 0.05; **, *P <* 0.001; ***, *P <* 0.001; ns, not significant). **C**.–**E**. RF regression to generate SPAs (C), SMAs (D), and SIAs (E) of the Chinese-SPM cohort (*n* = 246). In all the plots, LOESS regression was applied on samples with a 95% confidence interval in shadow. SCCs between the predicted values and the observed values were calculated. RF, random forest; SPA, skin phenotype age; CA, chronological age; SD, standard error; MAE, mean absolute error; SMA, skin microbiome age; SIA, skin integration age; SCC, Spearman correlation coefficient; LOESS, locally weighted regression.

Next, we set out to integrate these ten SIPs into a proxy for such temporal dynamics. To this end, we conducted random forest (RF) (**Figure 2**A) and deep neural networks (DNNs) (Figure S4A) to regress ten skin phenotypic scores against the individuals’ chronological ages (CAs) in the Chinese-SP cohort (see Materials and methods). We chose RF regression for the following analysis because of its higher *R*^2^ (54.43% ± 3.37%) and lower mean absolute error (MAE = 5.48 ± 0.19 years) than those of the DNN regression (*R*^2^ = 20.81% ± 9.52%, MAE = 7.13 ± 0.98 years). The pore, pigment, and UV spot had the top importance in the RF regression (Figure 2A; Table S1), suggesting their representative roles in phenotypic aging, which was consistent with their strongest correlations with the CA (Figure S2). The predicted CA of a sample by RF regression was then designated as the skin phenotype age (SPA) of that sample. SPA could be used to estimate the skin phenotypic state and compare the state between individuals: if one had a younger SPA, his or her skin phenotypic state tended to be in a state of general people with younger CA. To test the gender effects on SPA, we trained and predicted SPA using female samples, and then applied the trained model to the male samples (Figure 2B). Owing to that the trained model was based on the skin phenotypes of female samples, if the predicted SPA of a male was older than that of a female with the same CA, the skin state of the male would be considered “older” than that of the female. We found that in the 20–65 CA range, males had older SPAs than those of females (Mann–Whitney–Wilcoxon test; *P <* 0.05), with the disparity widening before CA of 50 (Figure 2B). These results were consistent with those obtained when training the model with the male samples and applying it to the female samples (Figure S4B).

Beyond the skin age index based on skin phenotypes (SPA), we also established skin age indices based on skin microbial species [skin microbiota age (SMA)] and their combination with the phenotypes [skin integration age (SIA)], respectively, using the samples in the Chinese-SPM cohort (see Materials and methods). Considering the gender effects on SIPs, only female samples were recruited and analyzed in the Chinese-SPM cohort. The SPA showed *R*^2^ = 68.68% ± 11.73% and MAE = 3.35 ± 0.46 years (Figure 2C), and the SMA showed *R*^2^ = 52.81% ± 11.09% and MAE = 4.52 ± 0.28 years (Figure 2D), and the SIA showed *R*^2^ = 71.11% ± 5.84% and MAE = 3.22 ± 0.29 years (Figure 2E). *Moraxella osloensis*, *Streptococcus gordonii*, and *Streptococcus parasanguinis* were most representative in microbial aging (Table S1). Taken together, these three indices could be used to characterize the skin’s holistic state related to skin aging, in both phenotypic and microbial aspects, as well as unveil the key phenotypes and microbes in aging.

### Correlations between skin microbial composition and skin imaging phenome

**Figure 3:**
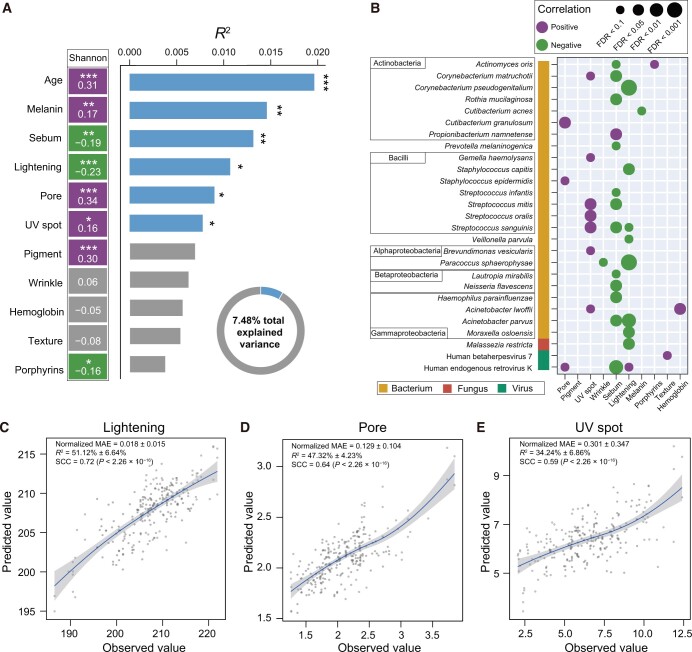
Correlations between skin microbial composition and skin imaging phenome **A**. The left squares show the SCCs of Shannon diversity at the species level with age and SIPs. The right bar plot shows the associations of the β diversity with age and SIPs, determined by PERMANOVA with 9999 permutations. *, *P <* 0.05; **, *P <* 0.01; ***, *P <* 0.001. **B**. Correlations of microbial species with SIPs were tested using MaAsLin analysis. **C**.–**E**. Predicted values of the SIPs were generated by performing RF regression on skin microbial species against the observed values of the three SIPs, lightening (C), pore (D), and UV spot (E). LOESS regression is applied to these samples with a 95% confidence interval in shadow. The normalized MAE was defined as the mean value of errors between paired observations divided by the corresponding observed value. SCCs between the predicted values and the observed values were calculated. PERMANOVA, permutational multivariate analysis of variance.

To investigate the correlations between skin microbial composition and SIPs, we first performed MetaPhlAn3 on 246 skin metagenomes of the Chinese-SPM cohort to obtain the microbial species profiles. We then calculated the α diversity (quantified by the Shannon diversity) and its Spearman correlation coefficients (SCCs) with the ten SIPs (**Figure 3**A; Table S2). Our results showed that the pore (*r* = 0.34) and pigment (*r* = 0.30) levels exhibited the highest positive correlations with α diversity (FDR < 0.1, Spearman correlation test). Conversely, SIPs such as lightening (*r* = −0.23) and sebum (*r* = −0.19) levels displayed the most notable negative correlations with α diversity (FDR < 0.1, Spearman correlation test). Moreover, we also tested the correlations between the β diversity (quantified by the Bray–Curtis distance at the species level) and the SIPs by using permutational multivariate analysis of variance (PERMANOVA) (Figure 3A; Table S2). Melanin (*R*^2^ = 1.5%) and sebum (*R*^2^ = 1.3%) levels emerged as the primary SIPs contributing to variations in β diversity. It was noted that the impact of age on the skin microbiome was also an undeniable factor, bearing the highest correlations with both α and β diversity. The collective influences of the SIPs and age accounted for a substantial 7.48% of the variability within the β diversity.

Next, we investigated the correlations between the single species and the SIPs, by using MaAsLin analysis with age adjusted. Our results showed that sebum and lightening levels were negatively correlated with many microbial species from multiple kingdoms, such as *Corynebacterium matruchotii*, *Staphylococcus capitis*, and *Streptococcus sanguinis* from bacterium, *Malassezia restricta* from fungus, and human endogenous retrovirus K from virus (FDR *<* 0.1, MaAsLin) (Figure 3B; Table S3). The UV spot level was positively correlated with *Streptococcus* spp. including *Streptococcus mitis*, *Streptococcus oralis*, and *S*. *sanguinis* (FDR *<* 0.1, MaAsLin). These findings collectively indicate that specific SIPs are intricately linked to the skin microbial diversity and composition.

### The skin microbial potential in predicting skin imaging phenome

Given the strong correlations between skin microbial composition and SIPs, our investigation proceeded to examine the microbial capability of predicting the SIPs. To this end, we performed RF regression on the microbial species against the observed value of the ten SIPs (Figure S5). Our analysis revealed that except for the porphyrin level, the remaining nine SIPs could be effectively predicted by the microbial species, with the normalized MAE ranging from 0.018 to 1.091. Microbial species demonstrated their highest predictive capability with regard to the lightening level (normalized MAE = 0.018 ± 0.015, *R*^2^ = 51.12% ± 6.64%) (Figure 3C), followed by the pore level (normalized MAE = 0.129 ± 0.104, *R*^2^ = 47.32% ± 4.23%) (Figure 3D) and the UV spot level (normalized MAE = 0.301 ± 0.347, *R*^2^ = 34.24% ± 6.86%) (Figure 3E). These results highlight the substantial potential of microbial species to serve as effective predictors of the SIPs. While the underlying mechanisms driving this predictive capability remain unknown, these results further validate the robust correlations existing between the skin microbiome and SIPs.

### The role of skin microbiome in shaping skin imaging phenome

We next set out to determine how skin microbiome interplays with the SIPs. To this end, we first mapped 4,511,788 metagenomic genes to the Kyoto Encyclopedia of Genes and Genomes (KEGG) database to obtain the KEGG ortholog (KO) abundance profile. Subsequently, we calculated the α diversity (quantified by the Shannon diversity) and the β diversity (quantified by the Bray–Curtis distance) based on the KO abundance profile, and found that these functional diversity measures were evidently correlated with the SIPs (Figure S6; Table S2). Pore (*r* = 0.32), melanin (*r* = 0.14), and pigment (*r* = 0.21) levels were positively correlated with the functional α diversity, while sebum (*r* = −0.14), porphyrin (*r* = −0.20), and lightening (*r* = −0.32) levels showed negative correlations (FDR < 0.1, Spearman correlation test). These findings mirrored the correlations between species-level α diversity and SIPs. Additionally, age was also a significant factor influencing both functional α and β diversity. The combined effects of SIPs and age accounted for a notable 12.59% of the observed variability within functional β diversity, among which sebum levels (3.1%) and pore size (2.5%) emerged as primary contributors. These findings coherently convey that SIPs such as sebum and pore levels hold strong correlations with microbial diversity at both taxonomic and functional levels.

**Figure 4:**
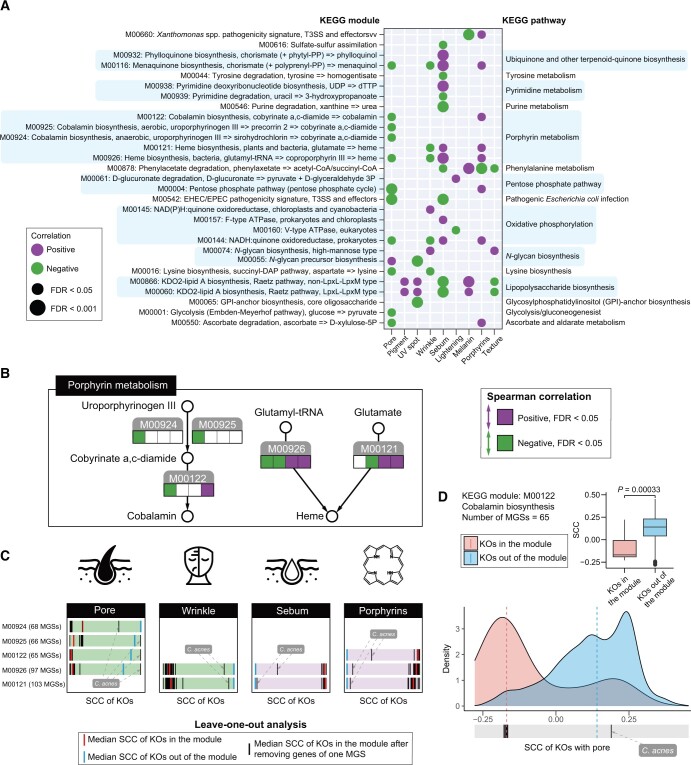
The role of skin microbiome in shaping skin imaging phenome **A**. The correlations of KEGG modules and each of the SIPs. **B**. KEGG schematic map of porphyrin metabolism pathway. The circles represent metabolites in the pathway. The KEGG modules of the pathway are colored in gray, and the four squares below represent the correlations of the modules with four SIPs including pore, wrinkle, sebum, and porphyrins (from left to right). The squares colored in purple/green refer to a positive/negative correlation with FDR *<* 0.05. **C**. Leave-one-out analysis to determine the MGSs that drove the correlations of the KEGG modules with four SIPs. For each bar, the horizontal axis represents the SCC of KOs with a specific SIP. The bar is colored in purple/green, which refers to the positive/negative correlation between the given module and the SIP, as indicated in (B). The red short line represents the median SCC between KOs in the given module and the SIP, while the blue short line represents the median SCC between the other KOs out of the module and the SIP. The black short line represents the median SCC between KOs in the given module and the SIP, after excluding the genes of one MGS. The length of each bar has been normalized. **D**. Density plot showing SCC between KOs in/out of the module M00122 and pore. The red dashed line represents the median SCC between KOs in the module and the pore, while the blue dashed line represents the median SCC between the other KOs out of the module and the pore. The box plot shows the differences in SCC between these two groups. Statistical significance was tested using the Mann–Whitney–Wilcoxon test. The black short line represents the median SCC between KOs in the given module and the SIP, after excluding the genes of one MGS. The removal of the genes of the MGS classified as *Cutibacterium acnes* could lead to the largest change in the correlations between the module M00122 and the pore.

Grouping KOs into the KEGG modules, we then delved into the correlations between these functional modules and SIPs. Owing to that a module contained multiple KOs (Table S4), the significance of the correlations was determined by if SCCs of the SIP with the abundances of KOs in the given KEGG module were significantly higher or lower than all the other KOs out of the module [25]. We identified a total of 29 modules that were evidently correlated with at least one of the ten SIPs (FDR *<* 0.05) (see Materials and methods) (**Figure 4**A; Table S5). Among them, we found that KEGG modules related to porphyrin metabolism were positively correlated with sebum and porphyrin levels, while negatively correlated with pore and wrinkle levels. The cobalamin biosynthesis (M00924, M00925, and M00122) and heme biosynthesis (M00926 and M00121) could produce porphyrins such as cobalamin and heme (Figure 4B), which might explain the positive correlations between these functions and the detected skin porphyrin levels. It is noteworthy that cobalamin deficiency has been reported to induce a lot of dermatological manifestations and dermatologic conditions [26]. These results suggest that the exogenous supplement of cobalamin and heme by the skin microbiome could potentially be linked to augmented sebum production and diminished pore and wrinkle formation.

Next, we sought to determine the skin microbes that drove the correlations of cobalamin and heme biosynthesis with the SIPs. To this end, we clustered genes into 109 MGSs (Table S6) and used leave-one-out analysis to determine the driving MGS. We found that when genes of MGSs classified as *Cutibacterium acnes* were removed, the SCCs between the modules of cobalamin and heme biosynthesis (M00924, M00925, M00122, M00926, and M00121) and SIPs were notably changed (Figure 4C; Table S7). For instance, as shown in Figure 4D, most of the KOs in the module M00122 were negatively correlated with the pore level, which was significantly different from the correlations between all the other KOs and the pore level (*P =* 3.3 × 10^−4^, Mann–Whitney–Wilcoxon test). After one MGS classified as *C*. *acnes* was removed, the median SCC of module M00122 had a notable increase of 0.36 and lost its negative correlation with the skin pore. These results indicate that *C*. *acnes* might modulate skin pores, wrinkles, sebum, and porphyrins through cobalamin and heme biosynthesis.

## Discussion

In this study, we first established three skin age indices based on phenomic and microbial features, highlighting the temporal dynamics of the skin imaging phenomes and microbiome. Moreover, we investigated the interactions between skin microbiome and skin imaging phenome from two facets. On the one hand, we identified the strong correlation patterns between skin microbial taxonomic/functional composition and SIPs. On the other hand, we provided a reservoir of functional modules and pathways that potentially modulated the SIPs.

Our findings shed light on that the skin imaging phenome might be shaped by collaborative effects of intrinsic aging and gender, as well as the skin microbiome. Using the Chinese-SP cohort, we demonstrated that age was the intrinsic factor in shaping skin imaging phenome. To eliminate gender effects, the Chinese-SPM cohort only encompassed female metagenomic and phenomic samples. Consistent with prior skin microbial studies based on 16S rRNA gene sequencing, our metagenomic analysis at the species level concurred with the notion that the α diversity of skin microbiome was higher among the elderly population, with discernible variations in the β diversity according to age [27]. Our study extended and validated these observations, demonstrating that not only microbial species but also functional diversity exhibited robust correlations with age and specific SIPs. When people got older, skin microbial α diversity would increase, which was linked to increased melanin, pore, pigment, and UV spot levels, and decreased sebum, lightening, and porphyrin levels. Moreover, the individual heterogeneity in the skin microbiome could be explained by age, sebum, pore, and melanin levels. With age adjusted, we still identified many species spanning kingdoms and functional pathways strongly correlated with specific SIPs, suggesting that besides intrinsic aging, skin microbiome may also play a role in shaping human skin imaging phenome.

Skin ages can help to clarify the controversial conception of a “healthy or not healthy” phenome or microbiome [28,29], by defining individual skin’s holistic state as most general people share at a certain CA. In general, people judge their skin phenotypic state by comparing a certain phenotypic score to a human-designed reference interval. However, skin state varies with chronological aging, and not all people share the same aesthetic preferences. For instance, we cannot regard an individual aged 60 years to have smooth and delicate skin, and it may be inappropriate to judge his/her skin state poor simply because the wrinkle score is out of the reference range. Additionally, not all people prefer lighter skin, so it is inappropriate to judge skin states poor due to the low lightening score. Moreover, there is even a lack of reference interval for defining a “healthy” skin microbiome [29], owing to its individual heterogeneity and high dimensionality [2,13]. However, these circumstances can be managed by skin age. Through regressing skin phenotypic and microbial features against people CAs in large cohorts, we can establish the skin age indices including SPA, SMA, and SIA. SPA represents what one’s skin looks like, based on ten SIPs. SMA represents the state of skin microbiota that cannot be directly observed in daily life but is closely linked to skin immunity and metabolism [2]. SIA integrates both skin phenotypic and microbial features, giving a relatively complete picture of the skin’s holistic state.

Besides skin age indices, this pioneering study has unveiled the potential of the skin microbiome in predicting skin imaging phenome. Notably, most SIPs such as sebum, pore, texture, hemoglobin, melanin, and lightening levels, could be predicted by skin microbial species profiles with an acceptable normalized MAE (less than 15%), among which the lightening level exhibited the lowest normalized MAE (1.8%). It should be noted that neither prediction of ages nor SIPs in this study is used to act as a model for prediction in practice, because such information could be easily acquired by other methods. The prediction of ages aims to build a proxy (skin age) to represent an individual’s skin phenotypic and microbial state, while the prediction of SIPs aims to reinforce the strong links between skin microbial species and skin imaging phenome.

In addition to enriching our understanding of the correlations between the skin microbiome and skin imaging phenome, our study has presented an invaluable reservoir of how skin microbiome adjusts the skin imaging phenome. Noteworthy examples include the modulation of pore, wrinkle, and sebum levels modulated by cobalamin and heme synthesis pathways, predominantly driven by *C*. *acnes*. This predominant member of the human skin microbes has been previously correlated with skin homeostasis, through lipid modulation, niche competition, and oxidative stress mitigation [30]. Our discoveries not only complement the multifaceted roles of *C*. *acnes* in shaping skin health but also provide a resource for understanding the underlying mechanisms by which skin microbiome modulates skin health.

This study also has limitations and prospects. First, our study established a comprehensive resource on the skin imaging phenome for Chinese people, which could be a reference for future studies. More samples from other countries could expand this resource to a global scale. Second, to control the gender effects, only female skin metagenomes have been investigated here. Male skin metagenomes might have different patterns and modulating effects on skin imaging phenome. Third, while our analysis has delved into the skin imaging phenome, it is important to acknowledge that skin phenome also encompasses physiological, metabolomic, and molecular features. Integrating data from these additional features could yield a more holistic blueprint of the intricate interplay between skin phenome and the microbiome. Lastly, the provided links between skin microbial functions and skin imaging phenome could serve as a foundational reference for further experiments.

## Conclusion

This study established three skin age indices by machine learning including SPA, SMA, and SIA as surrogates of phenotypic aging, microbial aging, and their combination, respectively. Moreover, this study presented the links between the skin microbiome and skin imaging phenome, including the strong correlations between compositional diversity and imaging phenome, the skin microbial potential in predicting imaging phenome, and the skin microbial functional potential in modulating specific imaging phenotypes. This study unveils the hidden roles of the skin microbiome in shaping skin appearance and healthy skin aging, providing new insights into the intricate skin microbiome–phenome interactions.

## Materials and methods

### Subject recruitment and sampling

Recruitment of the Chinese-SP cohort: 712 female and 286 male healthy volunteers aged 18–82 (29.08 ± 10.79) years were recruited from the general population living on the campus of Huazhong University of Science and Technology, Wuhan, China, between March and May, 2021. Recruitment of the Chinese-SPM cohort: a total of 246 females aged 20–60 (30.14 ± 9.21) years were recruited from the same campus between March and May, 2021. All subjects have provided informed consent. Medical and medication records of each subject were obtained through questionnaires. Subjects without any history of skin disorders and no antibiotic use in the past 6 months were accepted in this study. Each subject was asked to wash their face with tap water only and not to use any skincare products on their face 24 h before SIPs including lightening, sebum, porphyrins, texture, melanin, pore, pigment, wrinkle, hemoglobin, and UV spots were obtained by Chang’e Skin Detector (Catalog No. HH6800, Chang’e anti-aging, Wuhan, China) in a room at 25°C and 50% humidity without light [7]. Researchers wore sterile gloves each time they collected a sample with a sterile swab from the face cheek at room temperature with 50% humidity. To collect a sufficient amount of DNA, a sterile polyester fiber-headed swab, moistened with a solution comprising 0.15 M NaCl and 0.1% Tween 20, was used to swab a 4 cm^2^ section of the cheek repeatedly for a total of 50 times. Subsequently, the swab heads were broken off, placed in sterile centrifuge tubes, and stored at −80°C.

### Metagenome sequencing and processing

The microbial DNA was extracted utilizing the SolPure Tissue DNA Kit (Catalog No. D3312-01, Guangzhou Magen Biotechnology, Guangzhou, China) following the manufacturer’s protocols. Subsequently, the final DNA concentration and purity were assessed using a NanoDrop 2000 UV-Vis Spectrophotometer (Catalog No. NanoDrop2000, Thermo Fisher Scientific, Waltham, MA), and the quality of the DNA was confirmed through 1% agarose gel electrophoresis. For each sample, 500 ng of DNA was employed as input material in the DNA sample preparations. Sequencing libraries were produced using the Annoroad Universal DNA Fragmentase Kit V2.0 (Catalog No. AN200102-L, Annoroad Gene Technology, Beijing, China) and Annoroad Universal DNA Library Prep Kit V2.0 (Catalog No. AN200101-L, Annoroad Gene Technology) following the manufacturer’s recommendations, with index codes appended to associate sequences with individual samples. The clustering of the index-coded samples was performed on a cBot cluster generation system using HiSeq PE Cluster Kit v4-cBot-HS (Catalog No. PE-401-4001, Illumina, San Diego, CA) according to the manufacturer’s instructions. After cluster generation, the libraries were sequenced on an Illumina HiSeq X Ten platform (Illumina), producing 150 bp paired-end reads. Raw reads were initially trimmed to remove adapter and primer sequences. Reads containing low-quality bases (*Q* ≤ 19) constituting more than 50% of the total bases were eliminated. Reads containing “N” bases (bases not identified) representing more than 5% of the total bases were also discarded. Subsequently, reads were processed to exclude any read pairs in which at least one read aligned to the human hg38 reference genome using Bowtie2 (v2.4.5) [31]. The statistical information of metagenomic reads is available in Table S8. Metagenomic reads were assembled into contigs using MEGAHIT (v1.2.9) [32] with default parameters from the preset “meta-sensitive”. Open reading frames (ORFs) were predicted employing MetaGeneMark (v3.38) [33]. Predicted ORFs with a minimum length of 100 bp were clustered using cd-hit (v4.6.6) [34] with a sequence identity cutoff set at 0.95 and an alignment coverage cutoff set at 0.9. This process yielded a catalog of 4,511,788 non-redundant genes.

### Profiling of metagenomic samples

The abundance profiles for each catalog gene were generated using NGLess (v1.1.0) [35] as follows: (1) metagenomic reads were aligned to the non-redundant gene catalog using minimap2 (v2.14) [36]; (2) reads that successfully mapped to genes were filtered based on a length of 30 bp and a minimum identity of 95%; (3) the abundance of each gene was estimated by counting the number of short reads that mapped to it (in cases where a short read mapped to multiple genes, the abundance was distributed among those genes using the “count” function in NGLess, with the parameter “multiple” set as “dist1”); and (4) to enable comparisons between different samples, the abundance profiles were normalized based on the size of each library.

For the functional abundance profile, the catalog genes were annotated using the KEGG database (release 94.0) through KofamKOALA (v1.3.0) [37]. The resulting KOs were then mapped to the KEGG module annotation obtained from the KEGG BRITE database on May 5, 2022. The KO abundance profile was computed by aggregating the abundance of genes associated with each KO. The taxonomic abundance profile was obtained by MetaPhlAn3 (v3.0.13) [38] with default parameters based on metagenome reads. In total, we detected 822 species from 285 genera, 129 families, 59 orders, 29 classes, 14 phyla, and 3 kingdoms (bacteria, fungi, and viruses).

### Metagenomic species construction

Catalog genes that were detected in at least three samples were grouped together based on their co-abundance using the canopy algorithm (https://bitbucket.org/HeyHo/mgs-canopy-algorithm/src/master/) [39,40]. This process resulted in the identification of 2416 co-abundance gene groups (CAGs) showing strong correlations (Pearson correlation coefficient > 0.9). The canopy profiles were computed for each sample as the 75th percentile of the abundance across all genes within the respective CAG. Out of these, a total of 109 CAGs, each containing over 500 genes, were designated as MGSs. Taxonomic annotations were assigned to the MGSs through the following steps [39,40]: (1) catalog genes were taxonomically annotated by comparing their sequences to the National Center for Biotechnology Information Reference Sequence (NCBI RefSeq) database (downloaded on December 1, 2022), which encompasses 24,280 complete bacterial genomes, 21 complete fungal genomes, and 11,340 complete viral genomes, utilizing BLASTN with an E-value threshold of < 1.0 × 10^−5^; (2) for each gene, only the best alignments with a length of 100 bp or more were retained; (3) MGSs were assigned taxonomic classifications at the species, genus, or family levels if over 50% of their genes, including those with no matches, exhibited sequence similarity of 95%, 85%, or 75%, respectively; and (4) an MGS was categorized as a classified species when 80% or more of its genes were annotated to the same species.

### Establishment of the skin age indices

Skin age indices include SPA, SMA, and SIA, which were established by RF regression of skin phenotypic scores, microbial species relative abundances, and their combination, respectively, against the individuals’ CAs using R packages ranger (v0.13.1) and crossRanger (v0.1.0), as previously described [41]. Five-fold cross-validation was used in the RF progression. In brief, in each of the five iterations of the RF regression, four of five randomly divided sub-datasets were used for training, and the remaining one was used for the CA prediction. Five-fold cross-validation was used to calculate feature importance by the “permutation” mode of the function ranger of the R package ranger. In this process, the *R*^2^ of the five iterations was calculated. Additionally, the MAE between the predicted CA and the observed CA was calculated. The predicted CA of a sample was then designated as the SPA, SMA, and SIA of the sample, which was based on the CA with ten SIPs, microbial species, and their combinations, respectively.

To evaluate the effects of gender on skin age, the regression models were first trained and used for skin age prediction within a sub-dataset stratified by gender using five-fold cross-validation, and in each iteration, the trained model was also applied to the other sub-datasets, with the average of the predicted CAs from five iterations as the skin age of the other sub-datasets.

Besides using RF regression, the DNNs were also implemented to establish the skin age indices, by using Python 3.6 Keras library with TensorFlow backend. However, the performance of skin age models based on RF regression performed better than those based on the DNN regression. Therefore, the skin age models based on RF regression were used for the following analysis.

### Prediction of the SIPs by skin microbial species

Each of the ten SIPs was predicted by RF regressing skin microbial species against the observed scores of the SIPs, by using R packages ranger (v0.13.1) and crossRanger (v0.1.0). Five-fold cross-validation was used in the RF regression. In brief, in each of the five iterations of the RF regression, four of five randomly divided sub-datasets were used for model training, and the remaining one was used for the prediction of the SIPs. In this process, the *R*^2^ of the five iterations was calculated. Additionally, the MAE between the predicted scores and the observed scores was calculated. To make the MAE comparable between different phenotypic scores, we introduced a normalized MAE that was defined as the mean value of errors between paired observations divided by the corresponding observed score. This normalization procedure ensured a standardized assessment of prediction accuracy, thus enabling effective cross-phenotype comparisons.

### Statistical analyses

#### Linear regression to determine the associations between age and SIPs

Each of the SIPs was the independent variable and the age was the dependent variable. Statistical significance was defined as *P* < 0.05. To account for multiple hypothesis testing, the threshold for statistical significance was set at FDR < 0.1.

#### Mann–Whitney–Wilcoxon test to determine the significant differences between independent categorical data

For categorical data, samples were pooled into bins (age groups, female/male group, KOs in a module/KOs out of the module, *etc.*) and significance was defined as *P <* 0.05. To account for multiple hypothesis testing, the threshold for statistical significance was set at FDR < 0.1.

#### PERMANOVA to determine associations between microbiome profiles and SIPs

The associations of overall microbial composition with ten SIPs and age were determined by PERMANOVA with 9999 permutations at species and KO levels. In this analysis, the distance matrices were calculated by Bray–Curtis dissimilarities. Statistical significance was defined as *P* < 0.05.

#### MaAsLin analysis to determine associations between microbial species and SIPs

Age was adjusted in this analysis. Statistical significance was defined as *P* < 0.05. To account for multiple hypothesis testing, the threshold for statistical significance was set at FDR < 0.1.

#### Spearman correlation test to determine associations between microbial α diversity and SIPs

Microbial α diversity was quantified by the Shannon diversity at species and KO levels. The correlations between microbial α diversity and SIPs were calculated by the Spearmon correlation test with age adjusted. Statistical significance was defined as *P* < 0.05. To account for multiple hypothesis testing, the threshold for statistical significance was set at FDR < 0.1.

#### Associations between KEGG modules and SIPs

The relationships between KEGG modules and individual SIPs were assessed based on whether the SCCs of the phenotype scores with the abundances of KOs in the specified KEGG module were significantly higher or lower compared to the abundances of all other KOs. This assessment was conducted using the Mann–Whitney–Wilcoxon Test, with a threshold for statistical significance set at FDR < 0.1.

#### Leave-one-out analysis to identify driving MGSs

MGSs responsible for influencing the observed correlations between KEGG modules and each of the SIPs were determined through a leave-one-out analysis. This process involved iterating the calculation of KO abundance while excluding genes from a distinct MGS in each iteration. The impact of a particular MGS on a designated correlation was quantified as the alteration in the median SCC between KOs of the specified module and the SIP, when genes from the MGS were omitted from the analysis.

## Ethics statement

The Ethics Committee of Tongji Medical College, Huazhong University of Science and Technology, China (Approval No. S017), granted approval for all procedures involving human participants. In addition, informed consent was duly obtained from each participant.

## Supplementary Material

qzae040_Supplementary_Data

## Data Availability

The metagenomic sequencing data for this study have been deposited in the Genome Sequence Archive [42] at the National Genomics Data Center, Beijing Institute of Genomics, Chinese Academy of Sciences / China National Center for Bioinformation (GSA: CRA008646), and are publicly available at https://ngdc.cncb.ac.cn/gsa. All the skin phenome datasets and codes for the results of this study are available at https://github.com/MingyueCheng/Skin_paper_codes.

## CRediT author statement

**Mingyue Cheng:** Conceptualization, Methodology, Investigation, Resources, Software, Validation, Formal analysis, Data curation, Writing – original draft, Writing – review & editing, Supervision, Visualization. **Hong Zhou:** Methodology, Investigation, Resources, Formal analysis, Data curation, Writing – original draft, Writing – review & editing, Visualization. **Haobo Zhang:** Methodology, Investigation, Formal analysis, Visualization. **Xinchao Zhang:** Investigation, Formal analysis, Resources. **Shuting Zhang:** Investigation, Resources. **Hong Bai:** Investigation, Resources. **Yugo Zha:** Investigation, Resources. **Dan Luo:** Investigation, Resources. **Dan Chen:** Investigation, Resources. **Siyuan Chen:** Writing – review & editing. **Kang Ning:** Conceptualization, Visualization, Writing – original draft, Writing – review & Editing, Supervision, Project administration, Funding acquisition. **Wei Liu:** Conceptualization, Visualization, Writing – original draft, Writing – review & editing, Supervision, Project administration, Funding acquisition. All authors have read and approved the final manuscript.
