# Supplementary material for: Hidden Links Between Skin Microbiome and Skin Imaging Phenome
Source: Genomics Proteomics Bioinformatics. 2024 Jun 7;22(4):qzae040. doi: 10.1093/gpbjnl/qzae040 (PMC11849492; doi:10.1093/gpbjnl/qzae040)

Female (Age = 23 years)

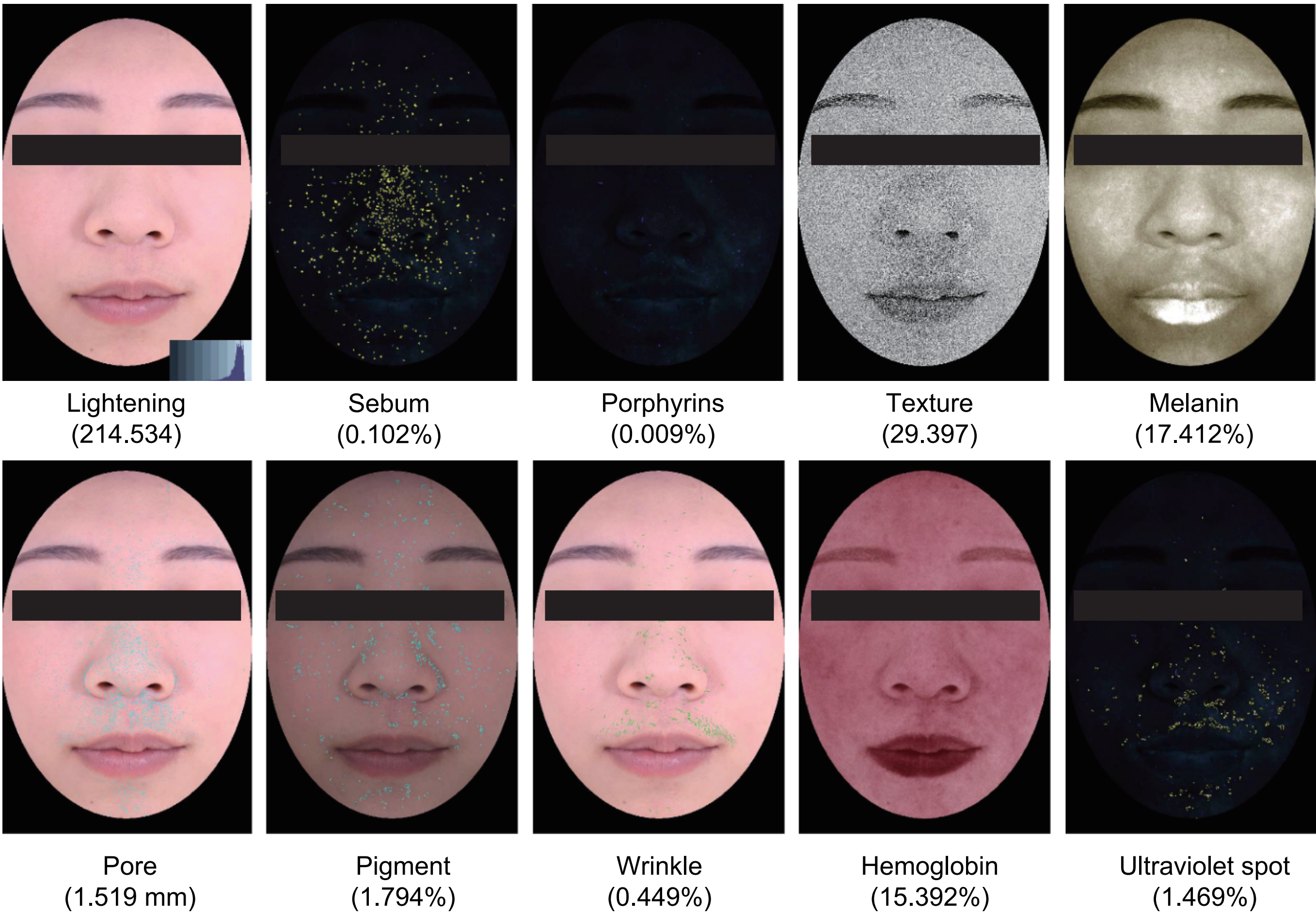

Male (Age = 23 years)

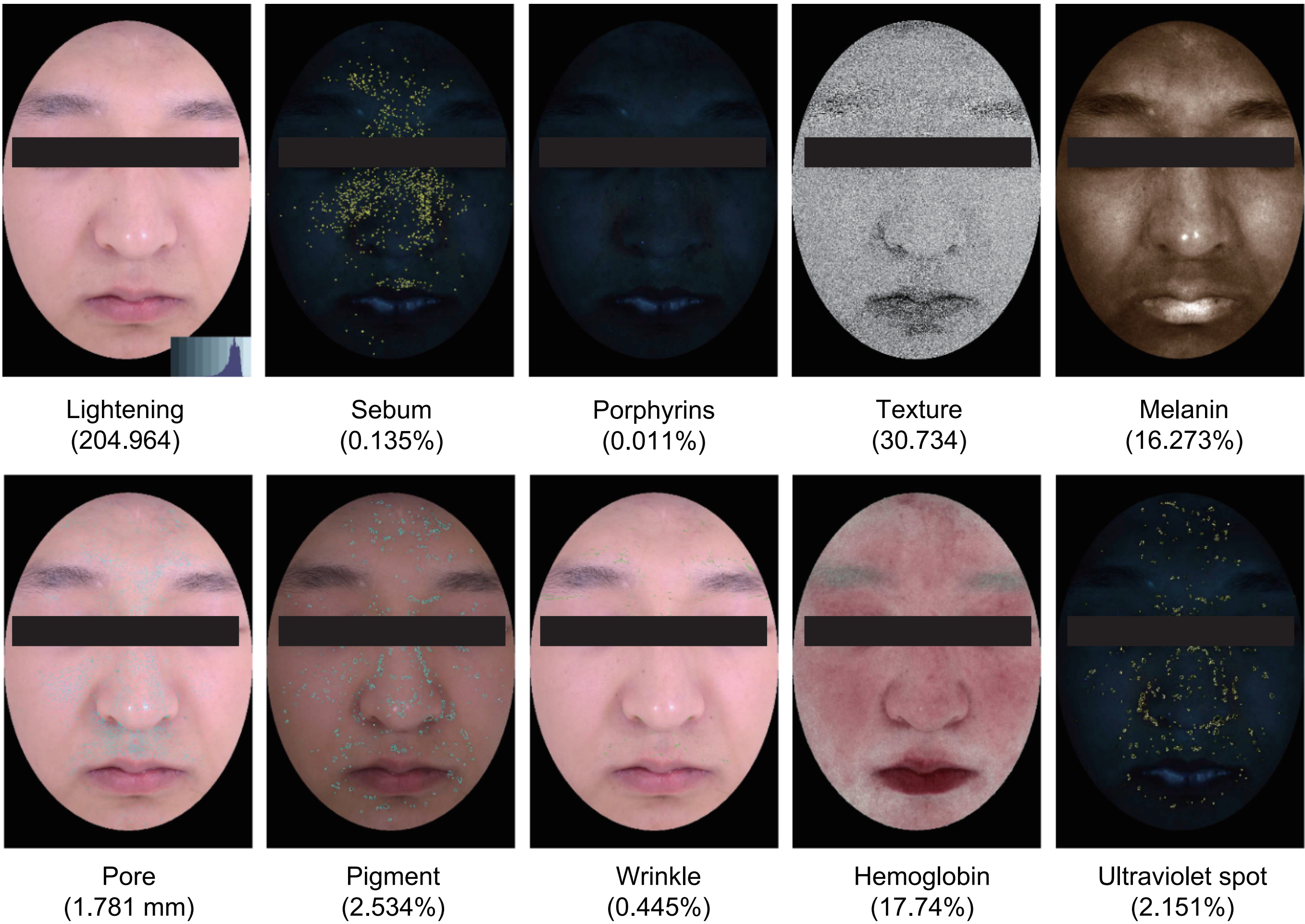

Supplement: qzae040_Supplementary_Data [file qzae040_supplementary_data.zip › Figure S1.pdf]
