# Supplementary material for: Hidden Links Between Skin Microbiome and Skin Imaging Phenome
Source: Genomics Proteomics Bioinformatics. 2024 Jun 7;22(4):qzae040. doi: 10.1093/gpbjnl/qzae040 (PMC11849492; doi:10.1093/gpbjnl/qzae040)
Supplement: qzae040_Supplementary_Data [file qzae040_supplementary_data.zip › Supplementary material captions.docx]

# Supplementary material

**Figure S1 An instance of skin imaging**

A total of ten SIPs were collected.

**Figure S2 Correlations between skin imaging phenome and age**

Linear regression between scores of each SIP and age in the Chinese-SP cohort, with dots colored according to the gender and 95% confidence interval band are shown in gray. BH, Benjamini–Hochberg method.

**Figure S3 Variations of skin imaging phenome grouped by gender and age**

**A.** Box plots showed SIPs in the Chinese-SP cohort across five age groups. **B.** Box plots showed SIPs in the Chinese-SP cohort across five age groups, divided by gender. Mann–Whitney–Wilcoxon test. *, *P <* 0.05; **, *P <* 0.01; ***, *P <* 0.001.

**Figure S4 Establishment of skin age** **indices**

**A.** DNN regression was used to generate SPAs of all the samples (*n* = 998) of the Chinese-SP cohort based on their SIPs. LOESS regression is applied with a 95% confidence interval in shadow. **B.** RF regression was used to generate SPAs of males (*n* = 286) of the Chinese-SP cohort based on their SIPs, and then applied the trained model of males on female samples (*n* = 712). LOESS regression of male and female samples is applied with a 95% confidence interval in shadow. Box plots and line charts show the differences in SPAs across five groups of CAs. The differences in SPAs reflect the differences in the skin phenotypic states between males and females. For all the box plots, statistical significance was tested using the Mann–Whitney–Wilcoxon test. SCCs between the predicted values and the observed values were calculated. *, *P <* 0.05; **, *P <* 0.001; ***, *P <* 0.001; ns, not significant. DNN, deep neural network.

**Figure S5 Skin imaging phenome predicted by skin microbial species**

In the Chinese-SPM cohort, predicted values of the SIPs were generated by performing RF regression on skin microbial species against the observed values of the SIPs. LOESS regression is applied to these samples with a 95% confidence interval in shadow. The normalized MAE was defined as the mean value of errors between paired observations divided by the corresponding observed value. SCCs between the predicted values and the observed values were calculated.

**Figure S6 Correlations between skin microbial function and skin imaging phenome**

In the Chinese-SPM cohort, the correlations of skin microbial KO composition with ten SIPs and age were determined by PERMANOVA with 9999 permutations. Spearman correlations of KOs Shannon diversity with ten SIPs and age were tested. *, *P <* 0.05; **, *P <* 0.01; ***, *P <* 0.001.

**Table S1 Feature importance in the establishment of skin age indices**

**Table S2 Correlations between skin imaging phenomes and microbial taxonomic and functional diversity**

**Table S3 Correlations between skin imaging phenome and microbial species**

**Table S4 Description of KEGG modules and KOs in this study**

**Table S5 Correlations between skin imaging phenome and KEGG modules**

**Table S6 Identification and annotation of the metagenome species**

**Table S7 Leave-one-out analysis to identify the driving metagenome species**

**Table S8 Statistical information of metagenomic reads**
