# Supplementary figures and images for: Hidden Links Between Skin Microbiome and Skin Imaging Phenome
Source: Genomics Proteomics Bioinformatics. 2024 Jun 7;22(4):qzae040. doi: 10.1093/gpbjnl/qzae040 (PMC11849492; doi:10.1093/gpbjnl/qzae040)

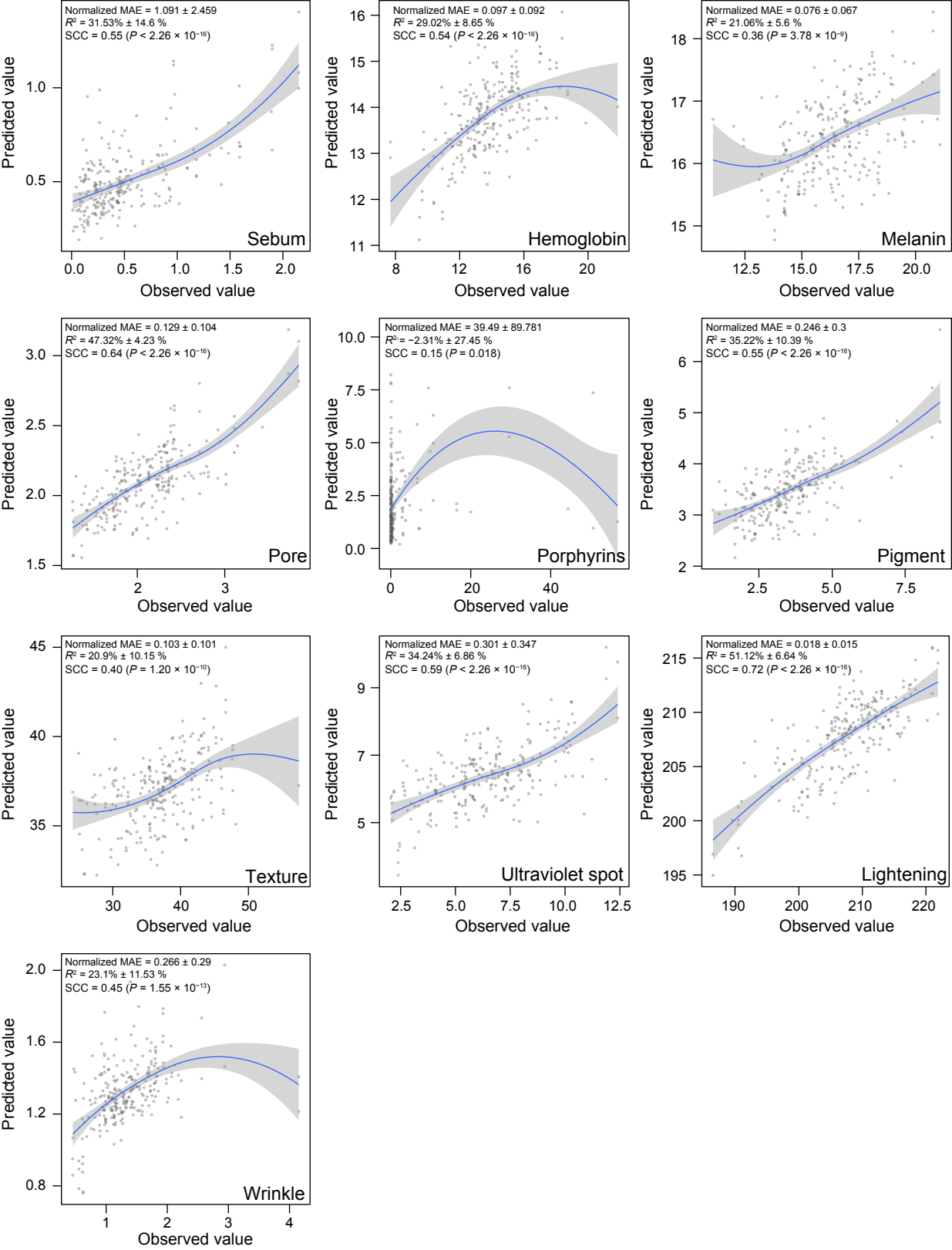

Supplement: qzae040_Supplementary_Data [file qzae040_supplementary_data.zip › Figure S5.pdf]

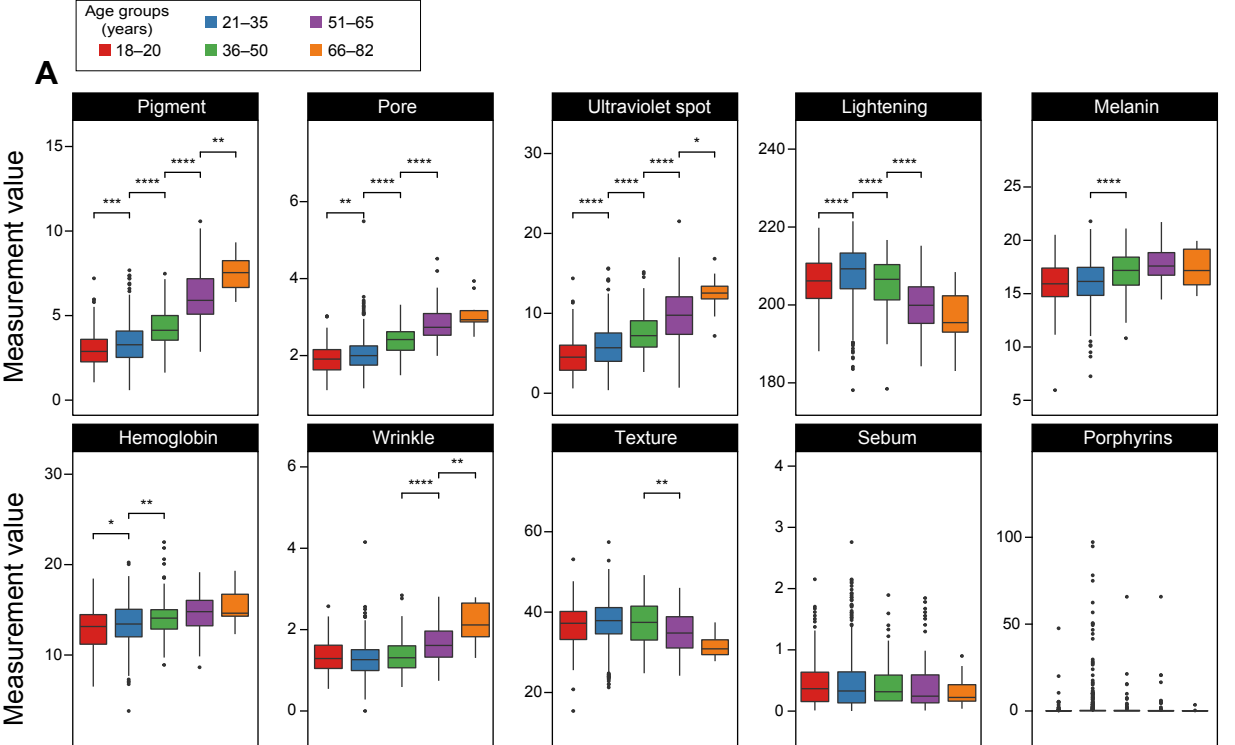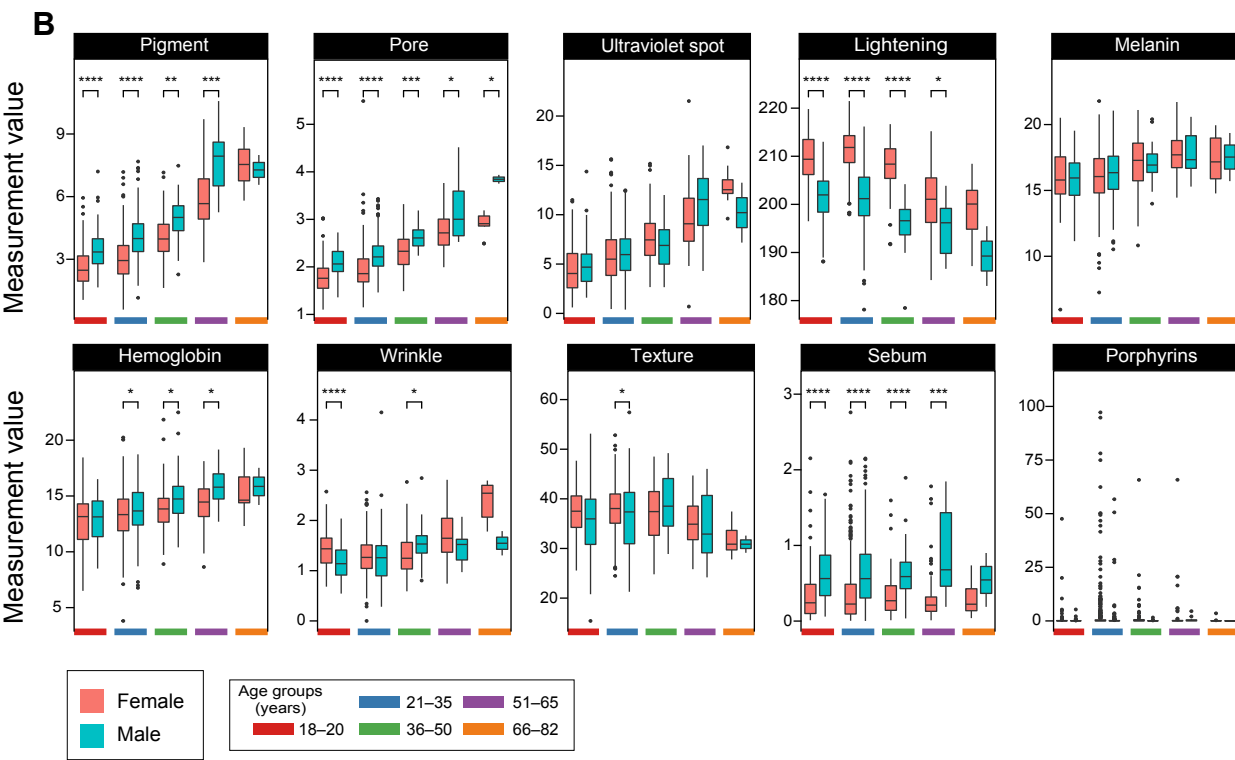

Supplement: qzae040_Supplementary_Data [file qzae040_supplementary_data.zip › Figure S3.pdf]

**A**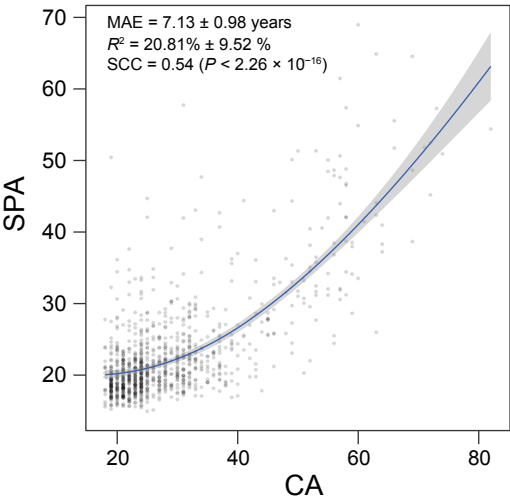**B**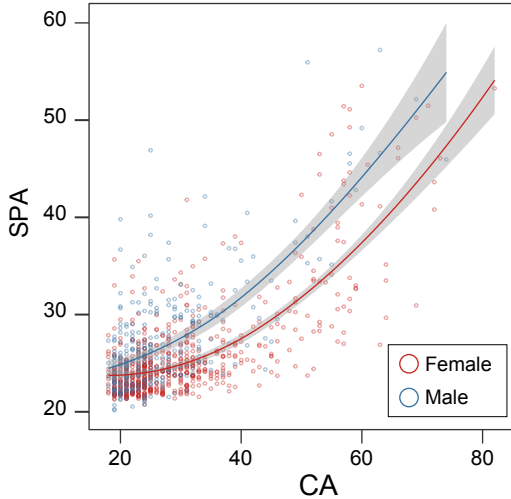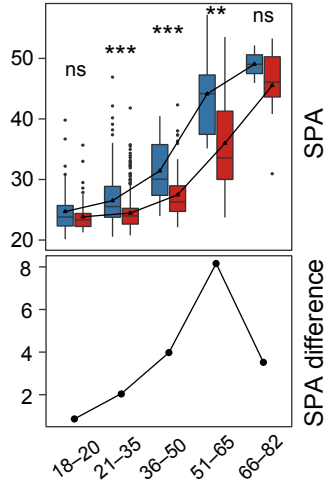

Supplement: qzae040_Supplementary_Data [file qzae040_supplementary_data.zip › Figure S4.pdf]

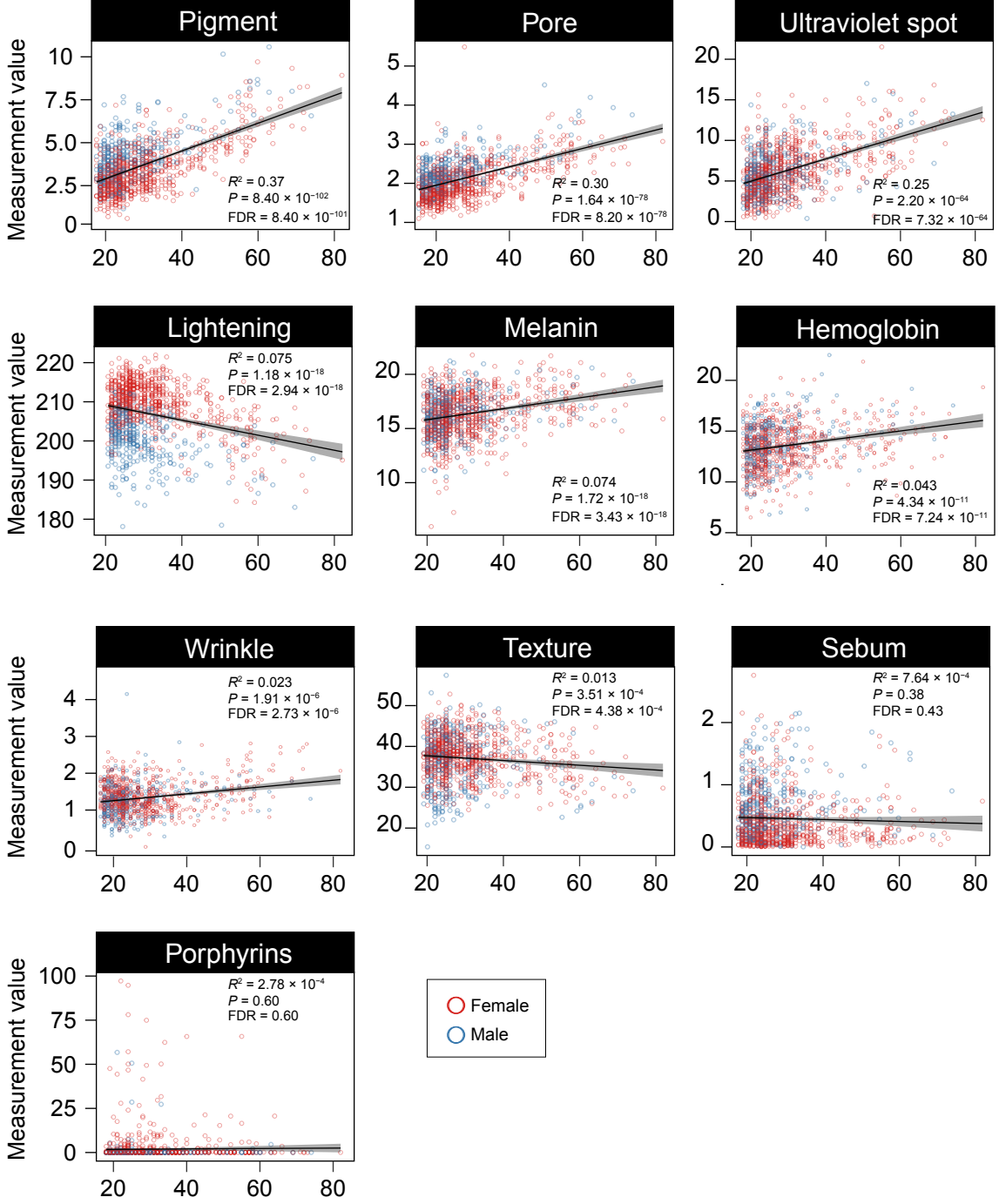

Supplement: qzae040_Supplementary_Data [file qzae040_supplementary_data.zip › Figure S2.pdf]

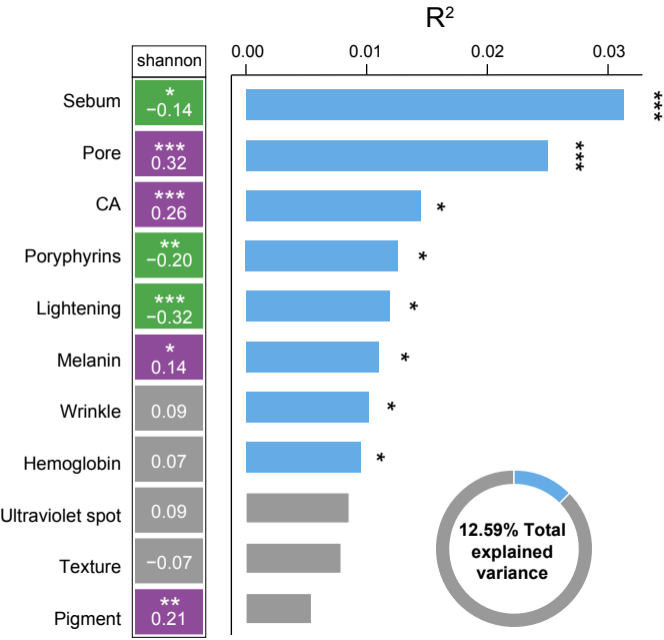

Supplement: qzae040_Supplementary_Data [file qzae040_supplementary_data.zip › Figure S6.pdf]
